# The work experiences and career development expectations of Chinese respiratory therapists: a descriptive qualitative study

**DOI:** 10.3389/fmed.2024.1452508

**Published:** 2024-08-29

**Authors:** Jianfeng Xu, Pengfei Cheng, Hangqing Yu, Niya Zhou, Meijuan Lan

**Affiliations:** Department of Nursing, The Second Affiliated Hospital of Zhejiang University School of Medicine, Hangzhou, China

**Keywords:** respiratory therapists, occupational experiences, qualitative research, career development, COVID-19 pandemic

## Abstract

**Background:**

Respiratory therapists (RTs) play a vital role in healthcare, specializing in the treatment and management of heart and lung conditions, particularly during the COVID-19 pandemic. Despite the importance of RTs, little attention has been paid to RTs in the Chinese health system. This study aimed to understand and describe the work experiences and career development expectations of RTs in China.

**Methods:**

This study utilized a qualitative research design and conducted semi-structured, in-depth, audio-recorded interviews with 16 RTs aged 28–40 years, purposively selected from six regions in mainland China from May to June 2024. Qualitative thematic content analysis was conducted to identify and group the themes that emerged from the discourse. Interviews were transcribed and analyzed using thematic analysis.

**Results:**

Four key themes were identified: (1) “Complex Career Motivation” delves into the career trajectories and role adaptations of RTs; (2) “Positive Career Feedback” explores feelings of job satisfaction and career accomplishments; (3) “Uncertain Career Predicament” sheds light on the negative impact of multidimensional career challenges; (4) “Demand-Driven Future Outlook” reflects RTs’ urgent expectations for professional advancement.

**Conclusion:**

The barriers and facilitators in the professional journey of Chinese RTs maintain a fragile balance, and the future development of the profession may determine whether they can persist in their careers. Healthcare managers and leaders should pay attention to the real needs of RTs, enhance their professional confidence, and adopt effective coping strategies to avoid the loss of human resources and promote the development of the professional team.

## 1 Introduction

Respiratory Therapists (RTs) are indispensable in the evaluation, treatment, and management of cardiopulmonary diseases (1). They collaborate closely with multidisciplinary teams to deliver comprehensive respiratory care across various clinical settings, including hospitals and home care environments (2). These professionals excel in diagnosing and managing critically ill patients, utilizing a range of skills to enhance treatment outcomes (3). RTs play a crucial role in facilitating early weaning from mechanical ventilation (4, 5), significantly reducing the risk of ventilator-associated pneumonia, and accelerating patient recovery (6, 7), thereby lowering medical costs (8). Particularly within the intensive care unit (ICU) setting, RTs enhance the cost-effectiveness of treatments by implementing personalized respiratory therapy programs, leveraging expert knowledge, and making independent decisions (9). Their interventions not only directly alleviate the financial burden on patients but also contribute significantly to the economic sustainability of the healthcare system. In chronic respiratory disease management, RTs offer a holistic approach encompassing diagnosis, treatment, education, and management (10). This comprehensive care not only improves patients’ quality of life but also equips them with enhanced self-management capabilities (11). Furthermore, RT interventions effectively reduce emotional distress, readmission rates, and post-discharge mortality risks (12–14). The work of RTs also eases the workload on medical staff and improves hospital operational efficiency (15). In essence, RTs play a vital role in enhancing patient outcomes and healthcare system efficiency, particularly in ICU care and chronic respiratory disease management. Their interventions have a profound impact on patient recovery and healthcare delivery effectiveness.

The multidimensional value of RTs in medical practice has been widely recognized and demonstrated (13, 16–18). In Europe, respiratory therapy as a child of the physical therapy professional treatment (2, 6). European respiratory society is responsible for the coordination of professional personnel training, and development of publishing education courses, aims to standardize the breath evaluation, treatment and follow-up of patients with respiratory system diseases required core knowledge, skills, attitudes and ability (19, 20). In North America, the United States employs an estimated 135,800 RTs, providing vital services nationwide (21). Similarly, Canada has a robust workforce of over 12,000 licensed RTs, each possessing extensive clinical expertise, comprehensive knowledge, and specialized skills in cardiopulmonary care (22). In Asian countries and regions, the development of the respiratory therapy profession varies. For instance, the Philippines, Singapore, and Taiwan have successively established respiratory therapy disciplines largely following the American model (19). Among them, Taiwan established a respiratory therapy department in 1973 and is one of the regions in Asia with more comprehensive organization and mature development of the discipline (20). In contrast, the development of respiratory therapy in Mainland China started relatively late. Despite 30 years of development, the number of respiratory therapists in Mainland China remains below 1,000 (20, 23). Given the vast population of China and the significant demand for respiratory care among patients with respiratory system diseases, the current number of RTs is evidently insufficient. This indicates that, in China, the clinical importance of RTs has not yet received widespread attention, and the development in this field remains suboptimal. The global outbreak of coronavirus disease 2019 (COVID-19) has underscored the pivotal role of respiratory therapy in clinical care and has further highlighted the exceptional contributions of RTs to healthcare teams (24). Concurrently, it has also exposed the human resource crisis and chronic underinvestment in the field of respiratory therapy in various countries (25). As the first country to report the COVID-19 virus, China faces unique challenges due to the relatively slow development of respiratory therapy, posing particular difficulties for RTs in treating patients with COVID-19 (26). A survey conducted in China revealed that each RT manages an average of 17–26 COVID-19 patients, directly engaging in crucial respiratory treatments such as lung-protective mechanical ventilation, prone position ventilation, pulmonary rehabilitation, airway management, and the transportation of critical patients (27). Furthermore, findings from a multi-center cross-sectional survey involving 593 RTs showed that among Grade A tertiary hospitals in provincial capitals of China, only 13.6% had established RT positions, with a mere 56.7% of these hospitals forming dedicated respiratory treatment teams. Regarding workload, 56.8% of RTs worked more than 40 h per week, 72.8% managed more than 10 inpatients daily, and nearly all (99.7%) were responsible for both inpatient and outpatient respiratory care (28). The statistics not only highlight the critical shortage of RTs in China but also poignantly illustrate the heavy workload shouldered by the few who are in service. This disparity between the scant number of RTs and the overwhelming demand underscores the urgent necessity for expanding the respiratory therapy workforce to adequately address the respiratory health needs of the vast Chinese population.

The stark contrast between a dire shortage of human resources and an overwhelming workload, coupled with the medical demands posed by severe pneumonia during the pandemic, has intensified the persistent professional challenges faced by RTs. These challenges encompass resource limitations, excessive workload, emotional burnout, inequitable treatment, and workplace bullying. Consequently, numerous RTs have encountered profound occupational fatigue and a heightened inclination to leave their positions (29–31). A qualitative study by Trachtenberg et al. (30) uncovered that even in the third year of the pandemic, RTs continued to struggle with negative emotions such as fear and anxiety, alongside inescapable physical and psychological stress. More alarming are the high rates of post-traumatic stress disorder (PTSD), depressive symptoms, anxiety, and stress observed among RTs during the pandemic (32). These occupational stressors and mental health issues have led some RTs to contemplate abandoning their dedicated careers (32, 33). The aftermath of the COVID-19 pandemic is expected to have enduring repercussions on the respiratory therapy profession, particularly concerning staff retention and recruitment. In addition to RTs nearing retirement, many have exited the profession during the pandemic due to the intense stress and demanding hours. This exodus, paired with a significant decline in enrollment in RT education programs and a dwindling number of students eager to pursue the profession, has precipitated a critical and perilous shortfall in the RT workforce, a trend that is projected to escalate in the coming years (34). This situation poses a significant threat to the long-term sustainability and stability of the RT workforce, especially in nations where the respiratory therapy profession is still emerging (35).

RTs in China remain a relatively small and often overlooked group, attributable to the nascent stage of respiratory therapy in the country. To date, Chinese researchers have predominantly employed quantitative research methods, particularly cross-sectional surveys, to gage the development status of RTs. However, these studies have seldom delved into the actual work experiences and career development aspirations of RTs. While quantitative research is valuable, it can be limited in exploring contexts and understanding the complexity of phenomena. In contrast, qualitative research offers a more nuanced perspective for examining the intricate details and deeper meanings of these experiences. It provides critical insights into how RTs navigate the challenges inherent in career development. Although previous qualitative studies have highlighted the work experiences of RTs in developed countries during the pandemic, our understanding of the specific professional experiences of RTs in developing countries in the post-pandemic era, especially in China where respiratory therapy is in an early, exploratory growth phase, is still limited. These experiences are inherently subjective and can be influenced by a multitude of factors, such as cultural diversity, the stage of professional development, and the level of policy support (36). In view of these considerations, this study adopts qualitative descriptive research methods to deeply explore the work experiences and career development expectations of RTs in China. The aim is to authentically represent their occupational realities, shed light on the professional dilemmas they encounter, and investigate the various factors that influence career development.

## 2 Methods

### 2.1 Design

This study is a descriptive qualitative research. The interview data were collected from a semi-structured interview conducted in Mainland China from May to June 2024. Thematic analysis was conducted following the steps of Braun and Clarke (37). This report adheres to the Consolidated Criteria for Reporting Qualitative Research (COREQ): a 32-item checklist for interviews and focus groups (38) (Supplementary file S1).

### 2.2 Setting and sample

Participants for this study were recruited from an ongoing respiratory therapy education program in Hangzhou, Zhejiang Province, China, which took place between May and June 2024. A purposeful sampling method was utilized to ensure a diverse group of participants, targeting individuals with a range of characteristics including gender, geographic region, and educational background. To capture a comprehensive spectrum of experiences, RTs with varying lengths of professional experience were deliberately selected to represent different career stages. As the principal researcher (JF) is associated with the hospital that hosted the continuing education program, the process of contacting potential participants was streamlined. The program head aided in identifying eligible participants who fit the established criteria. Once potential participants were identified, the principal researcher (JF) reached out via text message, extending an invitation and detailing the research objectives, participation requirements, and the informed consent process. After obtaining consent, further information regarding anonymity, confidentiality, and other pertinent research details was provided to ensure participants had a full understanding and were participating voluntarily. Interested participants were required to sign an informed consent form and provide basic personal information. An interview time was then scheduled accordingly. Eligibility criteria for participants included a minimum of 1 year of experience as RTs in a hospital setting and agreement to an individual, in-person interview. Individuals from healthcare management staff without direct clinical patient contact were excluded from the study. During the recruitment process, two RTs declined to participate due to scheduling conflicts. The sampling process continued until data saturation was achieved, which is indicated by the absence of new codes or themes emerging from the data. In total, 16 RTs were enrolled in the study.

### 2.3 Data collection

Building on an extensive literature review and following the methodology proposed by Kallio et al. (39), we initially developed an interview guide that included 15 potential questions (Supplementary file S2). A collaborative meeting was held with the research team to meticulously review and refine the guide. The focus was on eliminating ambiguous or leading questions and consolidating those that were likely to yield repetitive responses. Subsequently, we sought the expertise of a qualitative research methodologist and an ethics expert to assess the guide’s comprehensiveness and linguistic suitability for our study’s objectives. Based on their feedback, the interview guide was streamlined to nine open-ended questions, with adjustments made to the phrasing as required. The final version of the guide is presented in Table 1. Pilot interviews were conducted with the first two participants to validate the interview framework. These interviews included follow-up and exploratory questions designed to elicit nuanced responses and ensure clarity. Given the absence of further adjustments needed to the interview structure and the valuable insights obtained from the pilot interviews, these initial sessions were incorporated into the subsequent analysis.

**Table 1:** Interview guide.

Interview guide	
1.	Could you share the story of how you came to be in this profession?
2.	In your own words, what kind of work does a respiratory therapist do?
3.	What do you consider to be the most significant challenges in your role?
4.	What aspects of your work as a respiratory therapist bring you the most satisfaction or a sense of accomplishment?
5.	Is there a particular case or experience that has left a lasting impression on you that you’d like to share?
6.	Can you describe the dynamics of your collaborative relationships with physicians and nurses in a clinical setting?
7.	How would you assess the support and assistance provided by your leadership in terms of your professional development and work?
8.	Based on your work experience, what is your perspective on the career development of respiratory therapists?
9.	Is there anything else you would like to add?

All interviews were conducted by the same researcher (PF) to ensure methodological consistency. Verbal consent was obtained from participants to record the interviews, and the study’s purpose was reiterated prior to each session’s commencement. The interviews varied in length, ranging from 20 to 80 min. Participants were informed that they had the right to withdraw from the interview at any time without needing to provide a reason. To ensure a quiet, comfortable environment and to maintain consistency, all interviews were held in a designated meeting room, free from interruptions by unrelated individuals, thus safeguarding the privacy of the interviewees. Face-to-face, one-on-one in-depth interviews took place in a private setting. An audio recording device, the iFlytek Smart Recorder (model number: SR101), was utilized to capture the interviews, while field notes were taken to document non-verbal cues, such as facial expressions and body language of the participants. Recognizing that the researcher is an integral part of the research process, and despite thorough study and training in qualitative research by the researcher (PF), measures were taken to account for the potential impact of subjectivity on the research outcomes. This included the regular writing of memos during the study and the maintenance of a reflective journal to chronicle the researcher’s thoughts, questions, and any other pertinent ideas throughout the research process. Data collection and analysis were carried out concurrently and iteratively, with ongoing assessment of the sample size to ascertain when thematic saturation was achieved. The research team determined that after 14 interviews, the primary themes were preliminarily identified and data saturation seemed to be reached. Consequently, two additional interviews were conducted to verify this saturation, during which no new themes emerged. Therefore, data collection continued until no new themes appeared.

### 2.4 Data analysis

The interview recordings were transcribed verbatim by two principal investigators (PF and JF) within 24 h post-interview. They incorporated descriptions of non-verbal communication, such as silence, laughter, or sighs, at the appropriate points in the text. The investigators cross-checked each other’s work to ensure accuracy, and ambiguities were discussed and clarified with the interviewees when necessary, to enhance the authenticity of the transcription. All participants’ names were anonymized by replacing them with unique research identifiers. The transcripts were then returned to the participants for review and correction, ensuring their perspectives were accurately represented. Subsequently, they were imported into NVivo V12 software (QSR International) for data management and analysis. The content analysis adhered to the thematic analysis steps outlined by Braun and Clarke (37), facilitating a top-down approach to identify content and develop a profound understanding of the underlying phenomena. Using NVivo’s coding function, the two principal investigators (PF and JF) preliminarily labeled the data by meticulously analyzing the text sentence by sentence, applying coding tags for concepts, themes, or emotions. They iteratively refined and expanded these codes, informed by the concepts that emerged from the interviews. After generating preliminary codes, they categorized and organized them further, with each researcher creating a mind map and nodes to identify common patterns and recurring concepts. Discrepancies were resolved through discussions in team meetings until consensus was achieved. The final codes were summarized, grouped, and a matrix was constructed to analyze the underlying themes and subthemes. The original data were re-examined to verify the accuracy and completeness of these themes. The established themes and subthemes were then discussed in a reflective team meeting, where each theme was named and clearly defined. To prevent unintended interpretations, coding and theme generation were conducted in the original language (Chinese) and then translated into semantically equivalent English before drafting the report, ensuring that the translations maintained the original meanings and nuances.

### 2.5 Rigor

To ensure the rigor of our qualitative research, we adopted Lincoln and Guba’s criteria (40), focusing on credibility, transferability, confirmability, and dependability. Firstly, the principal investigators (PF and JF), both holding master’s degrees in nursing, possess the requisite capability and training to conduct research, having undergone systematic qualitative research study during their postgraduate education. Additionally, as practicing RTs, their dual roles facilitated the establishment of trust with participants, fostering an environment conducive to open expression and enabling a deeper understanding of participants’ backgrounds and experiences. To enhance the transferability and representativeness of our data, participants were selected from diverse geographical, economic, and demographic backgrounds. Following data collection, interviews with two additional RTs were conducted to verify thematic saturation. Confirmability was addressed through the use of participants’ quotes to support each theme, with results verified by the research team. Utilizing investigator triangulation in data analysis, including transcription, node establishment, coding, and theme generation, ensured diverse perspectives were considered. To mitigate personal bias, organized materials were reviewed by interviewees for accuracy. Finally, to enhance reliability, the interview guide underwent review by an ethics expert and the research team, and meticulous records were maintained using NVivo software, encompassing interview recordings, observation notes, reflective journals, and coding processes.

### 2.6 Ethical considerations

The study received approval from the ethics committee of the institution where the first author is affiliated (approval number: I2024585). Participants were fully informed about the study procedures, and it was made clear that their participation was entirely voluntary. Written informed consent was secured from the participants prior to initiating data collection. They were assured that they could withdraw from the study at any time without incurring any negative repercussions. Confidentiality was strictly observed throughout the study, with access to the audio recordings and transcripts restricted solely to the research team.

## 3 Results

### 3.1 Demographic characteristics

A total of 16 RTs participated voluntarily in the study, comprising 7 males and 9 females. Their ages ranged from 28 to 40 years, with professional experience in respiratory therapy spanning from 2 to 10 years. Among the participants, 2 had completed a dedicated respiratory therapy program, 3 had pursued studies in rehabilitation medicine during their undergraduate education, while the remainder had backgrounds in nursing. These participants were sourced from different regions across mainland China, with two individuals holding master’s degrees and the remaining 14 possessing bachelor’s degrees. Further details regarding participant characteristics are provided in Table 2.

**Table 2:** Demographic characteristics of participants (*N* = 16).

Participants	Sex	Age	Degree level	Educational background	Years of experience	Specific regions
R1	Male	36	Master	Nursing	7	Zhejiang
R2	Female	39	Bachelor	Nursing	5	Guangdong
R3	Female	31	Bachelor	Nursing	8	Xinjiang
R4	Female	31	Master	Nursing	3	Tianjin
R5	Male	35	Bachelor	Nursing	2	Zhejiang
R6	Female	31	Bachelor	Nursing	9	Xinjiang
R7	Female	29	Bachelor	Rehabilitation	9	Beijing
R8	Female	40	Bachelor	Nursing	9	Shanghai
R9	Female	31	Bachelor	Nursing	7	Hebei
R10	Female	30	Bachelor	Rehabilitation	6	Fujiang
R11	Male	31	Bachelor	Nursing	2	Zhejiang
R12	Male	32	Bachelor	Respiratory Therapy	9	Sichuang
R13	Male	28	Bachelor	Nursing	6	Hunan
R14	Male	35	Bachelor	Nursing	5	Hunan
R15	Male	31	Bachelor	Rehabilitation	10	Zhejiang
R16	Female	28	Bachelor	Respiratory Therapy	5	Shanghai

### 3.2 Results of the analysis

**Figure 1:**
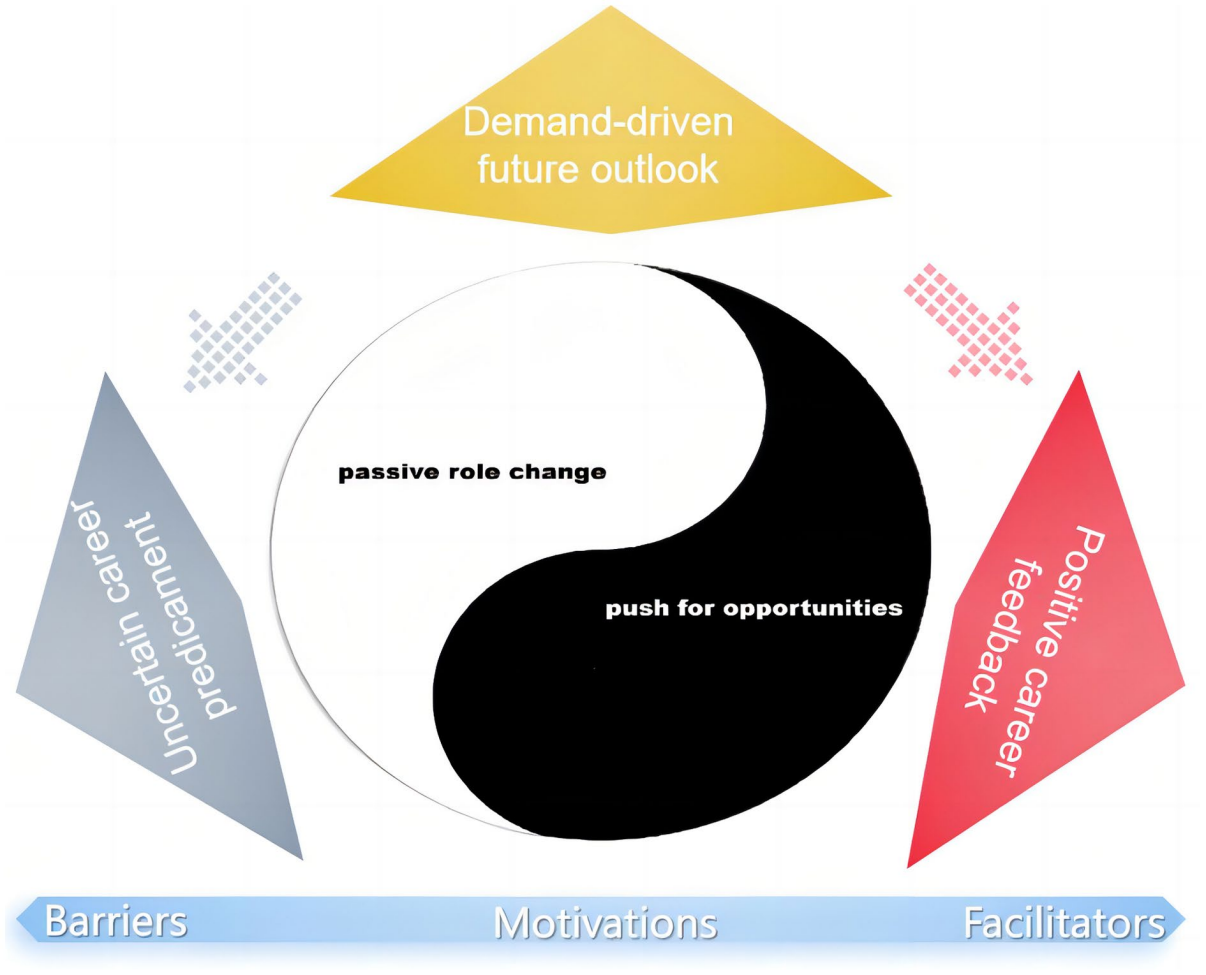
Thematic factors influencing the career development of RTs.

Four main themes and nine sub-themes emerged from the study data (the detailed coding tree is provided in Supplementary file S3). The first theme, titled “Complex Career Motivation,” delves into the career trajectories and role adaptations of respiratory therapists. Sub-themes within this theme include “Passive role change” and “Push for opportunities.” The second theme, “Positive career feedback,” explores feelings of job satisfaction and career accomplishments, encompassing sub-themes such as “Personal career achievement” and “Team interaction effectiveness.” The third theme, “Uncertain career predicament,” sheds light on the negative impact of multidimensional career challenges faced by respiratory therapists. Sub-themes within this category include “Bear Multiple Pressures,” “Career development confusion,” and “Value of profession questioned.” Lastly, the fourth theme, “Demand-driven future outlook,” reflects respiratory therapists’ urgent expectations for professional advancement, including sub-themes such as “widespread urgent appeal” and “Educational advancement” (Figure 1).

### 3.3 Theme 1: complex career motivation

This theme delves into the career trajectories and role adaptations of Chinese RTs. A portion of participants entered the field as a result of hierarchical personnel reassignments, engaging in a passive role transition in response to external directives imposed by the management. In contrast, another segment of individuals, driven by a dissatisfaction with their original profession or considering the significance of professional education in career shaping, proactively chose to join the ranks of RTs.

#### 3.3.1 Passive role change

When healthcare administrators recognize the conflict between the importance of RTs in clinical practice and the lag in professional education that leads to a talent resource mismatch, selecting trustworthy employees from within the institution for training or recruiting graduates from related disciplines capable of undertaking respiratory therapy responsibilities, such as those from the fields of rehabilitation medicine or nursing, becomes a compromise solution and a common choice for administrators.

At that time, I was a nurse in the intensive care unit. Suddenly I was informed by the leader that I was transferred, in fact, I still listen at a loss. (R14)

Although this adjustment may be significantly different and mismatched with the major they studied during their college years. But employees within medical institutions often do not have the right to refuse and must follow organizational instructions to transition to new roles. This often leaves participants feeling passive and helpless.

They (hospital administration) felt that someone with a rehabilitation background could also work in pulmonary rehabilitation, and that led to me being assigned to work as a respiratory therapist. Although it was not what I expected, I had to obey the arrangement, after all, I had to survive. (R15)

#### 3.3.2 Push for opportunities

Certainly, sometimes the choice of this profession is also a third path that had to be taken at the time, and it is even filled with rationality. Especially for participants who are from a nursing background, taking on the role of a respiratory therapist is to some extent a result of fatigue and a desire to disengage from nursing work. These reasons motivate them to compete and pursue this emerging profession more vigorously and proactively. Becoming a respiratory therapist represents an opportunity to alter their career trajectory.

Every night shift 12 hours, at least 2 times a week, my body can not tolerate, if I do not succeed, I will definitely resign, so I frequently apply to the nursing department to request a transfer to work. (R2)

The sentiment of wanting to leave the nursing profession is further amplified within the group of male nurses.

Which man would willingly commit to a lifelong career as a male nurse in the ICU? The expansion of the respiratory therapist team offered me a chance. (R11)

This means I can shed the label of ‘male nurse’; the status of a respiratory therapist sounds more respected, and I am grateful for the second chance. (R5)

For participants who have obtained a degree in respiratory therapy, the pursuit of a career within this discipline represents a logical and congruent decision that aligns with their academic training, professional ambitions, and personal ethos.

Many of my classmates did not aspire to become respiratory therapists after graduation. They felt like it was just about following standard procedures, which to them seemed similar to nursing, where there wasn’t much room for personal creativity. But, you know, I’ve never thought about it that way myself. (R16)

### 3.4 Theme 2: positive career feedback

Participants reported that the positive feedback they received during periods of close engagement with patients or their families significantly enhanced their sense of accomplishment in their critical roles of life-saving and healing. Moreover, knowing that their advice was taken seriously and integrated into a comprehensive treatment plan made participants more inclined to share their insights in a supportive environment.

#### 3.4.1 Personal career achievement

Observing patients regain health and experience improved respiratory function through one’s assistance is a direct and most pronounced means by which participants derive a sense of professional accomplishment.

The patients were really struggling with their lung conditions, but thanks to our hard work, they were finally able to get off the ventilator and move out of the ICU. It’s a huge win for us. (R7)

Receiving recognition and respect from patients and their families further amplifies this professional achievement through external validation.

I was totally surprised and touched when she (the patient’s family member) gave me a bunch of celery as a token of gratitude. You have no idea how precious vegetables were at that time (During the COVID-19 pandemic)… (R4)

Positive external feedback not only underscore the critical importance of RTs but also serve as a motivational catalyst for continual self-improvement. Ongoing acquisition and mastery of novel technologies facilitate professional advancement. This pursuit inherently engenders a sense of achievement.

I realize my own gaps when I first run into patients with extracorporeal membrane oxygenation. Then I hit the guidelines and bone up on new stuff to help ease their symptoms. In the process, I’ve leveled up too. (R12)

#### 3.4.2 Team interaction effectiveness

When participants have greater say in team collaboration and their suggestions from a respiratory therapy perspective are considered and adopted in clinical decision-making, they feel trusted and respected.

Four years ago that tank truck explosion accident, a large number of burn and explosion injuries of patients with rapid and dangerous changes in condition… Every multidisciplinary consultation listened to our advice, and the patient’s outcome improved, so everyone was very satisfied with us. (R1)

This sense of being valued can inspire them to offer help and support with greater enthusiasm.

Nurses seems to be more respectful to us and more dependent on us … When a patient’s problem is solved, the nurses will praise and admire us, which makes me happy to attend clinical consultations in these departments. (R13)

### 3.5 Theme 3: uncertain career predicament

The participants unanimously agreed that they are filled with uncertainty about their future career development. Sometimes when the issues cannot be resolved smoothly, or when they choose to retreat, postpone, or give up as a strategy, the difficulty of taking steps in adversity weakens their determination for the future.

#### 3.5.1 Bear multiple pressures

The unique respiratory therapy demands brought on by the pandemic have further exposed a catastrophic shortage of RTs; the initial cohort of RTs endured the impact of medical overcrowding, suffering both physically and psychologically, and felt deeply tormented. One participant recalled a difficult moment in their outbreak:

After the zero-COVID policy was lifted, we were hit with a tsunami of COVID-19 patients being admitted to the hospital. It was just the 14 of us trying to manage over 200 patients on ventilators. We barely got to rest for less than six hours a day. I’ve never been this exhausted in my life… (R13)

Participants also faced emotional distress and psychological peril, with the pandemic experience sending them spiraling into moral distress.

Is God trying to wipe them (Elderly COVID-19 patients) out? Every day, so many bodies are taken out of the ICU. It’s just devastating, and I feel so powerless… (R4)

During a pandemic painful experience even further cause physical discomfort. The female participant went on to explain the experience:

That day I watched the nurse do the cadaver care. Ever since then, I couldn’t eat meat again. I don’t know why… this state lasted for nearly 4 months. (R4)

#### 3.5.2 Career development confusion

Participants once believed that the pandemic would be a turning point for their career development, but it seems that after the pandemic, the situation has become even more obscure, and their careers have been marginalized once again. Participants feel besieged by a multitude of doubts. For the most part, the biggest question is who I am?

It’s tough to explain what our role is because the responsibilities in our job aren’t clearly defined. (R15)

Doctors don’t want to do it, and nurses can’t handle it. On a positive note, we’re like the indispensable piece of the puzzle. But to put it bluntly, we feel like the extra third wheel. (R1)

Participants emphasized, in addition to the lack of a clear career orientation, promotion and development path chaos also make them very confused.

We are like officially unrecognized underground lovers, and although I studied respiratory therapy in Taiwan, I now have to take another certification as a therapist in order to advance my career. (R16)

The emphasis placed on them during the pandemic once offered participants a ray of hope and strengthened their confidence. However, with the end of the pandemic and the sudden drop in clinical demand, they have gradually felt the disappointment and anger of being forsaken, leading to even greater confusion about the future.

We’ve weathered the pandemic together with the country, society, and hospitals, but now it feels like we’re being cast aside, back to square one—it’s really disheartening. (R6)

All the rosy promises of career advancement during the pandemic now seem to have turned out to be bad check. (R8)

Hope followed by disappointment is the real chilling. It seems like this profession really has no future. My confidence in the development of my career is fragile and unstable, just like a bubble. (R9)

#### 3.5.3 Value of profession questioned

The scrutiny and questioning from external sources are not only disheartening but may also lead to profound doubts about the significance of themselves careers. Unfortunately, the trivialization of participants’ professional values often comes from their immediate superior leaders, who have a clear cognitive bias toward the critical role that participants play in clinical practice. As a result, participants were often forced to take on some additional, non-professional work. Not only does this practice increase their workload, but more importantly, it diminishes the clinical value and professional standing of RTs and dilutes the recognition and influence they deserve within the medical team.

I’m not a secretary, but they keep dumping all this stuff on me—team-building, public outreach, party-building activities… It’s driving me up the wall. (R7)

… Or because managers can’t see our economic effectiveness, we are now considered unnecessary by our colleagues (doctors and nurses). (R15)

Moreover, there exists a considerable discrepancy between the efforts exerted by the participants and the corresponding rewards they receive. This incongruity not only erodes their job satisfaction but can also precipitate deep-seated doubts regarding their career path decisions.

It feels like we’re being robbed of the financial rewards we deserve for our hard work. If this situation continues, will our professional labor be inferior to cheap labor, and where will the value of our work be placed? (R13)

Subtle family dynamics can similarly undermine participants’ affirmation of their professional value and diminish their subjective performance in terms of occupational costs. This internal struggle often leaves them feeling disheartened and vexed.

Every time traditional festivals like the Mid-Autumn Festival or Chinese New Year roll around, they start complaining about how my job doesn’t pay much and how I’m never around. It really gets to me every time I hear it. (R10)

I often hear the older generation say, ‘Just quit your job and stay home to take care of the kids.’ It’s really hurtful, you know? (sighs) Is a woman’s worth only in raising children? (R6)

### 3.6 Theme 4: demand-driven future outlook

For the participants, nothing is more pressing than receiving adequate recognition and attaining professional certification at the national level. This is regarded not only as a fundamental building block on their career development journey but also as essential for securing greater flexibility and a wider array of development opportunities in the future, particularly within the domains of clinical professional education and academia.

#### 3.6.1 Widespread urgent appeal

Participants commonly feel that their professional contributions are not given the recognition and acknowledgment they deserve from the top down. One of the main challenges they face is the lack of a unified, nationally recognized certification system, which restricts their potential for development in clinical practice.

We urgently recommend that the national level should promptly carry out the professional certification for our field. We cannot remain in a gray area indefinitely. (R15)

Within the medical institutions they serve, the participants often find themselves in a paradoxical situation: despite their critical contributions, they are frequently overlooked by administrators, yet they deeply desire recognition for their efforts.

Hospital top-level decision-makers do not value us, and naturally, there is no investment in our profession. It’s been a constant state of waiting for support that never comes, and endless disappointment has become the norm. (R4)

#### 3.6.2 Educational advancement

In addition to the need for formal professional certification and recognition from management, RTs look forward to an educational system and academic exchange that are essential for the long-term sustainable development of their profession. They hope that by promoting the advancement of education and academia, they will not only be able to continuously improve their professional skills and knowledge level but also engage in in-depth academic exchanges with peers to jointly promote the development and innovation in the field of respiratory therapy.

I think the way we’re doing pre-job training and continuing education after we start working is pretty unstandardized, and honestly, it’s just not enough. (R1)

To really grow this profession in the future, we’re gonna need a solid education system in place at the college level, including both undergrad and grad programs. Sadly, we’re pretty much starting from scratch with that. (R15)

From what I know, there’s only one university in the whole country that takes grad students for respiratory therapy, and the number is pretty small. Their graduates pretty much have this exclusive little club going on, which isn’t ideal. (R16)

The academic sphere, while not their forte, was a subject that resonated deeply with the participants. They harbored a profound concern and a yearning to excel, despite feeling marginalized in their capabilities within this domain. Their emotional investment in the academic field was palpable, reflecting a strong desire to bridge the gap between their aspirations and their perceived shortcomings.

Working in a hospital, it’s a must to apply for research projects and publish a substantial amount of papers to get promoted. We’re good at doing the work, but conducting research? We’re out of our depth there. (R3)

The take home message is, academic chats help us keep our clinical practices top-notch, ensuring safety and effectiveness, while also leveling up our own expertise and skills. (R7)

## 4 Discussion

This study is the first to employ qualitative descriptive research methods to delve deeply into the work experiences and career development expectations of Chinese RTs. Through this research, we have gained a profound insight into the career motivations, achievements, challenges faced by this group, and their expectations for future career development. The study found that Chinese RTs have gone through a dynamic struggle filled with challenges and opportunities in their career development process. Their growth is influenced by a combination of factors that both promote and hinder their progress. Specifically, proactive career motivation and positive job feedback are key factors that encourage participants to maintain an optimistic attitude toward career development. Conversely, passive career choices and the challenges they face act as barriers that hinder their positive experience in career development. At the same time, The expectations for the future, based on current demands, stand at a critical crossroads. Participants are observing and waiting to see how this factor evolves over time. One extreme possibility is that these demands and expectations will gradually be transformed into new internal assets, thereby offsetting past deficiencies. The other possibility is that the inability to achieve effective displacement of these demands in times of adversity will lead to a deepening sense of disappointment in professional development.

This study uncovers the complexity of professional motivation among RTs and finds a correlation with McClelland’s Human Motivation Theory, particularly in terms of achievement and affiliation motivations (41). Individuals with a high need for achievement tend to set challenging yet attainable goals, enjoy self-improvement, and prefer autonomy in career decisions, making them more likely to actively pursue a career as RTs when opportunities arise (42). Whether transitioning actively or passively from their original organizational roles to become RTs, the disruption of social relations and work interactions necessitates a reevaluation of their needs. RTs with varying affiliation motivations exhibit diverse professional sentiments. Although the research highlights the multifaceted nature of RTs’ professional motivations, no motivations closely related to the need for power were identified. This may be attributed to the limited recognition and respect for the professional status of RTs in China, thereby constraining their ability to ascend to leadership positions within medical institutions through this profession. Given the diversity in motivations for becoming a respiratory therapist, managers should adopt differentiated strategies. For individuals who actively choose this path, managers should prioritize their career development and self-actualization needs. This includes offering continuous professional training, opportunities for further education, participation in seminars, and professional certifications to foster their growth. Providing increased responsibilities and decision-making authority allows them to assume leadership roles or contribute significantly to important projects, thus satisfying their achievement motivation (42). For those who did not initially choose to become RTs, managers should employ a more inclusive approach. This begins with establishing trust, understanding their personal goals and career expectations, and helping them find meaning and satisfaction in their work. Open communication channels should be maintained to encourage feedback and suggestions. Implementing a fair and transparent performance management system ensures their contributions are acknowledged, providing avenues for improvement and development. These efforts contribute to team stability, enhance overall satisfaction, and foster loyalty (43).

Our research indicates that Chinese RTs derive positive reinforcement from personal career achievements and efficient team collaboration during their professional journey. This encouragement promotes their development and strengthens their career beliefs (44). However, in stark contrast, barriers such as occupational stress, confusion, and skepticism significantly outweigh these promoting factors for Chinese RTs. Hakvoort et al. (45) have highlighted that when the balance between career-promoting and hindering factors is disrupted, it can increase turnover rates and inhibit recruitment of new employees. This underscores the potential development crisis and sustainability risks facing the Chinese respiratory therapist team. Drawing on career development experiences from other industries, we recognize that expanding existing promoting factors and creating new ones are essential measures in team management (46). Addressing the challenges faced by Chinese RTs requires intervention across various domains. From a leadership perspective, several areas warrant attention, including optimizing work systems, clarifying research objectives, planning clear career paths, ensuring adequate rest periods, minimizing non-professional tasks, and advocating for fair economic rewards for employees, among others. It is important to note that removing hindering factors does not automatically translate into promoting factors but can alleviate dissatisfaction among RTs with their current circumstances (47). Therefore, eliminating obstacles that contribute to negative professional experiences represents the initial step in resolving these issues. Simultaneously, ensuring fair treatment, respect, emotional support, and access to psychological counseling are crucial. Moreover, regular recognition, rewards, enhancing professional status, and clarifying role value, coupled with assisting in formulating career development plans, are vital to ensuring RTs perceive a clear growth trajectory within their organization.

The COVID-19 pandemic has starkly exposed the human resource crisis within China’s healthcare system, while also highlighting the pivotal role and potential value of RTs, prompting a reassessment of the profession’s legitimacy and recognition. Specifically, against the backdrop of the pandemic, in July 2022, China’s Ministry of Human Resources and Social Security formally included the RT profession in the national occupational classification for the first time, marking significant national recognition (28). However, compared to measures taken by other developed countries in response to the pandemic, China’s investment in respiratory therapy remains insufficient. The United States is expected to see a 13% increase in RT employment from 2022 to 2032 (21), while Canada is actively enhancing respiratory therapy education infrastructure, expanding collaborative medical teams, improving mental health support, and advocating for equitable professional status for RTs to meet future demand (22). In contrast, China lacks a national certification exam for RTs, hindering their ability to obtain formal professional qualifications. The relevant survey conducted by the Respiratory Disease Branch of the Chinese Medical Association found that only 24.5% of RTs in Mainland China are graduates with a major in respiratory therapy. 50.3% of RTs have obtained a nursing qualification certificate, 29.8% have obtained a qualification certificate as a practicing rehabilitation therapist, and 16.2% have a practicing physician qualification certificate. The absence of professional qualification certification for RTs not only affects the development of the respiratory therapy profession but also leads to confusion in the professional title promotion path for practitioners, restricting the career development of RTs and resulting in the loss of talent in the respiratory therapy profession. 82.4% of RTs believe that the lack of national qualification certification for respiratory therapy is the main reason for hindering the development of the respiratory therapy profession in China and causing the loss of professional talent (19, 23, 26). Moreover, only 13.6% of the tertiary hospitals in provincial capitals have RTs, and 17.0% of department heads believe that RTs can increase the department’s efficiency. The absence of top-level policies leads to neglect and indifference toward RTs by hospital management, which in turn constrains their professional growth within institutions (23, 26). Despite grassroots efforts that have yet to significantly influence national policy, RTs are pragmatically seeking ways to improve their working conditions. They particularly seek increased attention and support from hospital senior management, hoping for concrete commitments to enhance their working environment. Foremost among their expectations is ongoing professional training, essential for newcomers to acquire necessary competencies and for current practitioners to maintain their skills. However, China’s current training system lacks standardization, diversity, and stratification, thereby limiting comprehensive professional skill development among RTs. Furthermore, the shortage of qualified faculty not only impacts training quality but also impedes opportunities for ongoing learning and improvement (23). It is evident that the demand for RTs reveals complex realities and underscores urgent industry development challenges that require immediate attention.

Incorporating respiratory therapy into China’s medical education framework to cultivate proficient professionals tailored to the field is seen as a potentially effective long-term solution (28). A robust educational system encompassing both undergraduate and graduate programs aligns with industry expectations for RTs in China. Experience from developed countries highlights that a standardized and ongoing educational foundation catalyzes a qualitative leap, enhancing RTs’ capabilities and resilience within the demanding healthcare environment (48). This educational enhancement equips them with enriched clinical skills to manage diverse respiratory diseases and fosters deeper reflection on career development and academic pursuits. Over time, these trained professionals may evolve into qualified educators, nurturing the next generation of learners and fostering a positive cycle within the industry (49). It should be a cause for concern that only 12.9% of RTs in China hold a master’s degree or above, and 79.1% of RTs have a bachelor’s degree, indicating a scarcity of high-end RT talent (19, 26). A significant 34.7% of RTs have not published any scientific research papers since they started practicing, suggesting that the basic education and research capabilities of RT practitioners still need to be improved (23). The high-level development of China’s respiratory therapy profession is inevitably linked to the enhancement of research capabilities; conducting clinical scientific research is fundamental to the continuous development of the respiratory therapy profession. Therefore, future educational systems and talent training frameworks must focus on the professional and research capabilities of RTs. Moreover, fostering an open academic environment and maintaining international exchanges are crucial for China’s nascent field of respiratory therapy. Exchange programs with hospitals in North America and Europe have been instrumental in introducing and disseminating knowledge about RT practices in some Chinese hospitals. Institutions in provinces like Sichuan and Zhejiang, which pioneered respiratory therapy disciplines domestically, have also drawn upon and adapted Western methodologies and experiences (23). Sustaining these academic exchanges is vital for continuous updates in respiratory therapy theory, enhancing professional skills, promoting knowledge renewal, and broadening international perspectives among individual RTs (50). Furthermore, at the disciplinary level, international exchanges contribute to improving educational systems, increasing global influence, fostering talent cooperation, and breaking educational resource and academic exchange barriers (23). On a broader scale, these exchanges benefit China’s healthcare sector by informing and refining policies for RT training and management, fostering innovation, diversity, and progress across the medical industry, and enhancing China’s capacity to address global public health challenges such as COVID-19 amidst globalization (50).

The findings of this study hold significant practical and theoretical value for a deeper understanding of the work experiences and career development expectations of Chinese RTs. Firstly, these insights can provide invaluable reference for medical institutions and policymakers, helping them recognize the pivotal role of RTs in clinical healthcare and the urgent need to improve their working conditions and career development pathways. Secondly, the research underscores the urgency of establishing a nationwide professional certification system, which is crucial for promoting the standardization and professionalization of the RT profession. Furthermore, this study offers a case from an Eastern developing country, providing a valuable reference perspective for RTs in other developing countries. This helps facilitate understanding and communication of similarities and differences in cross-cultural contexts, enhancing international cooperation and exchange within the respiratory therapy profession. Lastly, the results of our study are of significant guidance to healthcare professionals and policymakers in designing tailored interventions for addressing burnout and human resource crises among RTs, strengthening individual career planning, and enhancing job stability. Through these measures, the career development of RTs can be more effectively supported, their contributions to the healthcare system can be increased, and patients can be ensured to receive high-quality respiratory therapy services.

This study acknowledges several limitations. Firstly, China’s economic regional disparities result in varying medical standards across different areas, which in turn affects the emphasis placed on RTs. Given that our research participants were primarily drawn from a specific continuing education program, with a higher concentration from the economically developed East China region, including areas such as Shanghai, Zhejiang, and Jiangsu, our data may be somewhat skewed toward regions with richer medical resources and superior professional care. Additionally, the COVID-19 pandemic may have influenced participants’ recollections, introducing potential biases. However, this retrospective reflection and review, particularly regarding work experiences during the pandemic, provide a unique opportunity to deeply uncover the genuine insights and emotions of the participants in the face of extraordinary crisis situations.

## 5 Conclusion

In conclusion, this study revealed their professional feelings in terms of career motivation, job satisfaction, career challenges, and future development expectations through in-depth analysis of the work experiences and career development expectations of Chinese RT. The results of the study not only provide an empirical basis to improve the working environment of RT, but also offer a foundation for their career development. Moreover, the study promotes the development of professional standards in respiratory therapy. In addition, it serves as an important reference for the standardization and specialization of the respiratory therapy profession. Future research should further explore how to improve the professional status and job satisfaction of RT through policy support and education and training.

## Data Availability

The original contributions presented in the study are included in the article/Supplementary material, further inquiries can be directed to the corresponding author.
