# Supplementary material for: The work experiences and career development expectations of Chinese respiratory therapists: a descriptive qualitative study
Source: Front Med (Lausanne). 2024 Aug 29;11:1452508. doi: 10.3389/fmed.2024.1452508 (PMC11390457; doi:10.3389/fmed.2024.1452508)

Supplementary File 3:Coding tree.

This figure includes a simplified version of the coding four including the most frequently described codes in the interviews


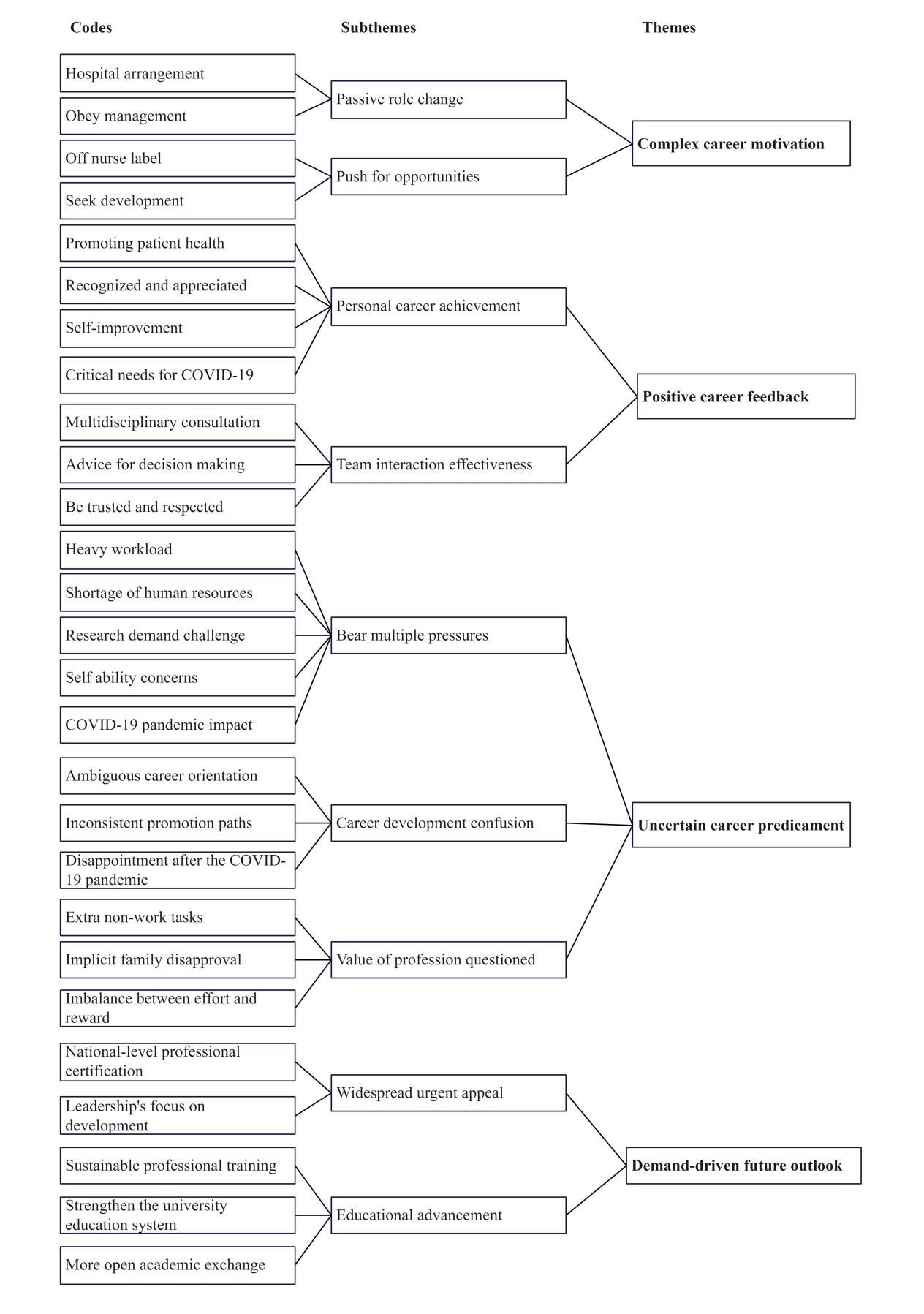

Supplement: Supplementary file 1 [file Data_Sheet_1.docx]
