# Supplementary material for: The work experiences and career development expectations of Chinese respiratory therapists: a descriptive qualitative study
Source: Front Med (Lausanne). 2024 Aug 29;11:1452508. doi: 10.3389/fmed.2024.1452508 (PMC11390457; doi:10.3389/fmed.2024.1452508)
Supplement: Supplementary file 2 [file Table_1.DOCX]

Supplementary File 1:Consolidated criteria for reporting qualitative studies (COREQ): 32-item checklist

| **No** | **Item** | **Guide questions/description** | **Page and heading** |
| --- | --- | --- | --- |
| **Domain 1: Research team and reflexivity** | | | |
| Personal Characteristics | | | |
| 1. | Interviewer/facilitator | Which author/s conducted the interview or focus group?  **- The first author** | **Page 5-6: Data collection** |
| 2. | Credentials | What were the researcher's credentials? *E.g. PhD, MD*  - **Master of Nursing** | **Page 6: Rigor** |
| 3. | Occupation | What was their occupation at the time of the study?  - **Respiratory therapist** | **Page 6: Rigor** |
| 4. | Gender | Was the researcher male or female?  - **Male** |  |
| 5. | Experience and training | What experience or training did the researcher have?  - **Educated in Motivational Interviewing** |  |
| Relationship with participants | | | |
| 6. | Relationship established | Was a relationship established prior to study commencement?  - **Yes** | **Page4:Setting and sample** |
| 7. | Participant knowledge of the interviewer | What did the participants know about the researcher? e*.g. personal goals, reasons for doing the research*  - **The participants were informed that the interview was for research** | **Page4:Setting and sample** |
| 8. | Interviewer characteristics | What characteristics were reported about the interviewer/facilitator? e.g. *Bias, assumptions, reasons and interests in the research topic*  - **Because of the interviewers background as a respiratory therapist it was under consideration throughout the entire study process** | **Page 6:Ethical considerations &Page 6: Rigor** |
| **Domain 2: study design** | | | |
| Theoretical framework | | | |
| 9. | Methodological orientation and Theory | What methodological orientation was stated to underpin the study? *e.g. grounded theory, discourse analysis, ethnography, phenomenology, content analysis*  - **Content analysis** | **Page 5:Data analysis** |
| Participant selection | | | |
| 10. | Sampling | How were participants selected? *e.g. purposive, convenience, consecutive, snowball*  - **Purposive sampling** | **Page4:Setting and sample** |
| 11. | Method of approach | How were participants approached? e*.g. face-to-face, telephone, mail, email*  - **Invitation with study information to participate was sent by text message** | **Page4:Setting and sample** |
| 12. | Sample size | How many participants were in the study?  -**16 participants** | **Page 6-7 :Results** |
| 13. | Non-participation | How many people refused to participate or dropped out? Reasons?  - **Two respiratory therapists declined participation due to scheduling conflicts during the recruitment process** | **Page4:Setting and sample** |
| Setting | | | |
| 14. | Setting of data collection | Where was the data collected? e*.g. home, clinic, workplace*  - **All participants were interviewed face-to-face, one-on-one in a private conference room** | **Page 4-5: Data collection** |
| 15. | Presence of non-participants | Was anyone else present besides the participants and researchers?  - **No** |  |
| 16. | Description of sample | What are the important characteristics of the sample? *e.g. demographic data, date*  - **demographic data** | **Page4:Setting and sample** |
| Data collection | | | |
| 17. | Interview guide | Were questions, prompts, guides provided by the authors? Was it pilot tested?  - **Yes, the guide is explained in the article** | **Page4-5:Data collection** |
|  |  |  |  |
| 18. | Repeat interviews | Were repeat interviews carried out? If yes, how many?  - **No** |  |
| 19. | Audio/visual recording | Did the research use audio or visual recording to collect the data?  - **The interviews was audiotaped** | **Page4-5:Data collection** |
| 20. | Field notes | Were field notes made during and/or after the interview or focus group?  - **Yes** | **Page4-5:Data collection** |
| 21. | Duration | What was the duration of the interviews or focus group?  - **From 20 to 80 minutes** | **Page4-5:Data collection** |
| 22. | Data saturation | Was data saturation discussed?  - **Discussion regarding informationspower** | **Page5:Data collection** |
| 23. | Transcripts returned | Were transcripts returned to participants for comment and/or correction?  -**Yes** | **Page5:Data analysis** |
| **Domain 3: analysis and findings** | | | |
| Data analysis | | | |
| 24. | Number of data coders | How many data coders coded the data?  - **3** | **Page 5: Data analysis & Page 6:Rigor** |
| 25. | Description of the coding tree | Did authors provide a description of the coding tree?  - **Yes** | **Supplementary File 3** |
| 26. | Derivation of themes | Were themes identified in advance or derived from the data?  - **Derived from data** | **Page 7: Results** |
| 27. | Software | What software, if applicable, was used to manage the data?  - **NVivo V12 software** | **Page 5: Data analysis** |
| 28. | Participant checking | Did participants provide feedback on the findings?  -  **No** |  |
| Reporting | | | |
| 29. | Quotations presented | Were participant quotations presented to illustrate the themes / findings? Was each quotation identified? e*.g. participant number*  - **Yes** | **Page 8 -13:**  **Results** |
| 30. | Data and findings consistent | Was there consistency between the data presented and the findings?  - **Yes** |  |
| 31. | Clarity of major themes | Were major themes clearly presented in the findings?  - **Yes** |  |
| 32. | Clarity of minor themes | Is there a description of diverse cases or discussion of minor themes?  - **No** |  |
