# Supplementary material for: The work experiences and career development expectations of Chinese respiratory therapists: a descriptive qualitative study
Source: Front Med (Lausanne). 2024 Aug 29;11:1452508. doi: 10.3389/fmed.2024.1452508 (PMC11390457; doi:10.3389/fmed.2024.1452508)
Supplement: Supplementary file 3 [file Table_2.DOCX]

Supplementary File 2:Initial interview guide

| The initial interviews include potential questions |
| --- |
| 1.You engaged in the work of beginner's mind, reason and karma is what? |
| 2.What do you think is a respiratory therapist's job? |
| 3.Do you have stress and burnout at work? |
| 4.What is your biggest challenge in your current job? |
| 5.In your opinion, what is one of the most accomplished things? |
| 6.In what ways do you think your job has improved your skills? |
| 7.Can you share the case that impressed you? |
| 8.What impact do you think your current clinical work has on your health? |
| 9.In COVID - 19 during a pandemic, what is your daily work life? |
| 10.What is the cooperative relationship with other colleagues (doctors and nurses) in the department in the clinical work? |
| 11.What kind of help and support has your leadership provided for your career development? |
| 12.Looking back on your career, what is the biggest gain or impression? |
| 13.What is your career plan for the future? |
| 14.What suggestions do you have for the development of respiratory therapists in China？ |
| 15.Is there anything else you would like to add? |
